# Supplementary material for: Malting—A method for modifying volatile composition of black, brown and green lentil seeds
Source: PLoS One. 2023 Sep 1;18(9):e0290616. doi: 10.1371/journal.pone.0290616 (PMC10473530; doi:10.1371/journal.pone.0290616)
Supplement: Attachment — Submitted filename: Reviewers comments.docx [file pone.0290616.s001.docx]

Dear Editor, Dear Reviewers,

Thank you most kindly for your time and your insight. We tried to improve the manuscript to the best of our ability and hopefully had not missed any of your remarks. Below are detailed comments (Line numbers are given for the manuscript with the changes marked)

*Malting can be a very promising areas in legume R &D. However, to establish the fact with evidences, some detailed analysis is required.*

*1.The authors must highlight the difference between malt extracts of cereals and legumes.*

Information about problems arising from the use of legume-based malts were added to the introduction [L. 47-54]

*2. Black, green and brown seems seedcoat colour of lentils. It is required to mention the cotyledon colour as well.*

Information about cotyledon colour was added. [L 63-65]

*3. What are the criterias of selecting these variety?*

These green and brown lentil varieties are most often grown in Poland (on the basis of the information from seed centers about how much of the seeds were sold in the latest years), while the black lentil variety is the only one grown currently in Poland.

*4. Some flowchart using infographics is better way to define methodology.*

Flowchart for the malting process was added (Figure 1)

*5. Line 32-34, "Malting is a process which is used primarily to modify grains of barley and the main reason of barley malting is increasing its enzymatic activity due to the generation and activation of various enzymes" Why in definition only barley is mentioned?*

Because over 90% of the malted material in the world is barley. This technique is typically used only for this kind of cereal, with small exception in malthouses producing wheat, rye and oat malt, but all the analyses, techniques and procedures are based solely on the barley grains.

*6. Introduction to be improved by adding more info. on malting in legumes.*

Data about malting legumes was added to the introduction [L 47-54]

*1. Abstract, Line 13-14, could you please explain? Try to end abstract on some conclusive note, highlighting the importance in future studies.*

Sentence was corrected (L.13-14).

*2. Introduction, Line 19-20, sentence seems to be irrelevant as it is not making any sense. Modify.*

Sentence was modified [L. 20-22].

*3. Results and discussion, A figure could be added to differentiate normal and malted lentils. A comprehensive approach explaining methodology and results in the form of a flow diagram could make this study more impactful.*

Flowchart of the malting procedure was added. (Figure 1). Additionally, figures showing changes in the total concentration of volatiles and pie charts showing changes in the composition of various groups of compounds in the volatilome of the lentil seeds and lentil malts were added (Figure 2, 3 and 4)

*5. Conclusions, Line 305-306, contradictory to abstract. Is there some typographic error or else?*

It was an error on our part, it was corrected in the abstract [L14-15].

*6. References, re-check for journal abbreviations and follow journal style as per guidelines.*

References and journal abbreviations were corrected.

*7. Grammatical errors, there is always a scope for improvement. Check whole article thoroughly and remove language errors to improve the overall quality.*

We have included corrections throughout the manuscript.

Reviewer #2:

*This paper is having a very routine information on biochemical constituents of malted lentil. The relevance of the results or the contribution of identified constituents for ensuring superior malting quality is not explained. In this context, it is not clear how the information is practically useful.*

The goal of the manuscript is to show, whether malting procedure could be used to change aroma of the lentils and to determine, whether it can be a useful method to increase or decrease concentration of various components, important in the food technology. We have included more information about malting legumes in the introduction [L.47-54], added diagram of the lentil malt production (Figure 1), included pie-charts showing changes in the composition of the volatilome in particular malts (Figure 2, 3 and 4), corrected various mistakes and added small part to the discussion (L. 221-223; L. 245-247).

*The statement in abstract section w.r.t the results is also not matching with the actual results.*

Our mistake was corrected in the abstract
