# Supplementary material for: Malting—A method for modifying volatile composition of black, brown and green lentil seeds
Source: PLoS One. 2023 Sep 1;18(9):e0290616. doi: 10.1371/journal.pone.0290616 (PMC10473530; doi:10.1371/journal.pone.0290616)
Supplement: Attachment — Submitted filename: Answer to the Reviewers.docx [file pone.0290616.s003.docx]

Dear Reviewer,

thank you for your kind words.

*“In the result and discussion section, the author should discuss why the total concentration of volatiles increased in green lentils but decreased in the case of black and brown lentils after the malting procedure.”*

We have added a small paragraph in the 3.1. section [Lines 193-198].

*In the method section, authors should add information related to the no. of replicates (biological and technical replicates) used in this study.*

The number of malting containers are mentioned in L. 110-113. Number of analyses is mentioned in L. 155-156.

*It is mentioned in the manuscript that in the malting procedure, lentil seeds are germinated for 4 days, 5 days, and 6 days. The authors should also incorporate the criteria for selecting the germination time.*

We used most typical malting period used by malthouses. More than 90% of malts are germinated for 120h and some of malt types can be malted for 4 or 6 days, however, this procedure is very unusual, because malting infrastructure (number of germination vessels: Saladin boxes or Galland drums) is installed in this number. As the malting is continuous through the whole year, significantly changing the germination time would be tremendously unpractical. We have added a short sentence (with the references) to the Methods section explaining number of germination days [L. 113-114].
