# Supplementary material for: Malting—A method for modifying volatile composition of black, brown and green lentil seeds
Source: PLoS One. 2023 Sep 1;18(9):e0290616. doi: 10.1371/journal.pone.0290616 (PMC10473530; doi:10.1371/journal.pone.0290616)
Supplement: Attachment — Submitted filename: Answers to the Editor.docx [file pone.0290616.s004.docx]

Dear Editor,

we tried to answer all your queries and hope that manuscript is up to the PLOS One standard.

Specific comments: *1.Language needs to be improved for more clarity like in line no. 20, 25 etc.*

We tried to clarify various sentences throughout the whole manuscript.

*2. In Line no. 23, it is written that lentil is warm season crop. However, lentil is cool season legume.*

We have written, that lentils can be grown in various climates - warm and cool alike, which is the truth. We never mentioned that lentils are warm season crop.

*3. Seeds are procured from pvt company. Can you mention that whether seeds are freshly harvested or store. If sored then how long it is stored?*

Seeds were stored prior the malting procedure. We mentioned storage time in the 2.1.1. Section

*4. Line no. 32, Authors defined malting in context of barley. Try to avoid that barley in definition.*

Barley was deleted from the definition.

*5. Line no. 61, please clarify and check cotyledon is red or orange?*

Yes, cotyledon is, of course, orange. We apologise for the mistake - it was a language mistake - in our country we call the orange lentils (cleaned and polished brown lentil) as the “red lentils”, despite their colour being, in fact, orange.

*6. Colour of fig. 1 is not professional. Keep it simple*

Colour was changed to the generic blue.

*7. Line no. 110 use eight in place of 8. Check and correct throughout MS. Digit less than 10 to be written in alphabets*

Corrected throughout the manuscript.
