# Supplementary material for: Malting—A method for modifying volatile composition of black, brown and green lentil seeds
Source: PLoS One. 2023 Sep 1;18(9):e0290616. doi: 10.1371/journal.pone.0290616 (PMC10473530; doi:10.1371/journal.pone.0290616)
Supplement: Attachment — Submitted filename: Reviewer comment.docx [file pone.0290616.s002.docx]

The manuscript entitled “Malting - a method for modifying volatile composition of black, brown and green lentil seeds” is well written. This study will widen the industrial use of lentils as malting improves their aroma. However, this manuscript may be accepted after the following minor revisions:

1. In the result and discussion section, the author should discuss why the total concentration of volatiles increased in green lentils but decreased in the case of black and brown lentils after the malting procedure. Some references should be added pertaining to the relation of seed colour with the concentration of volatiles.
2. In the method section, authors should add information related to the no. of replicates (biological and technical replicates) used in this study.
3. It is mentioned in the manuscript that in the malting procedure, lentil seeds are germinated for 4 days, 5 days, and 6 days. The authors should also incorporate the criteria for selecting the germination time.
